# A process-based approach to health-related quality of life as a “way of living”

**DOI:** 10.1007/s11136-023-03385-2

**Published:** 2023-04-01

**Authors:** R. P. Nolan, M. J. Sharpe

**Affiliations:** 1 Cardiac eHealth and Behavioural Cardiology Research Unit, Peter Munk Cardiac Centre, and Ted Rogers Centre for Heart Research, University Health Network, 6N-618NU, 585 University Avenue, Toronto, ON M2N 7A2 Canada; 2 Department of Psychiatry and Institute of Medical Science, University of Toronto, Toronto, Canada; 3 Department of Philosophy, Deakin University, Melbourne, VIC Australia

**Keywords:** Health-related quality of life, Well-being, Life aspirations, Eudaimonia, Hedonia, Stoicism

## Abstract

**Introduction:**

There is an historical initiative to establish common theoretical ground to support a framework for assessing health-related quality of life (HRQL). Our aim was to add to this effort with an analysis of theoretical/philosophical themes embedded in HRQL questionnaires and patient reports.

**Methods and Results:**

We reviewed recent developments in HRQL assessment. This included analyzing a representative sample of psychometric measures of HRQL to schematically summarize core theoretical/philosophical themes that are embedded in questionnaire items. This analysis indicated a state-based framework for HRQL that was characterized by themes of hedonic and eudaimonic well-being, and desire-satisfaction. In contrast, a review of patient reports of HRQL indicated a process-based framework where goal-directed activities aimed to secure aspirational life goals while striving to accept the reality of declining health. Given this difference in HRQL themes we used a meta-philosophical approach, based on Hadot’s idea of philosophy as a way of living, to identify a process-based theoretical framework for HRQL assessment that addressed patient-reported themes. The Stoic modification of eudaimonic well-being was examined where HRQL and well-being are viewed as a process (vs. state) aimed at transforming the experience of loss or grief in response to adversity through goal-directed activities/exercises (*euroia biou*, good flow in life). We then introduced a complementary research agenda for HRQL assessment that incorporates self-reported, goal-directed activities that are initiated or maintained to promote HRQL.

**Conclusion:**

A process-based approach to HRQL assessment may increase the spectrum of clinically relevant features that currently comprise operational measures of this patient-reported appraisal.

## Introduction

Health-related quality of life (HRQL) is advocated in policy statements as a primary endpoint to evaluate clinical interventions [1]. It is defined as a subjective evaluation of the effects of disease and health on one’s functional ability and capacity to live a fulfilling life [2]. HRQL assessments include single ratings [3] and multidimensional profiles that span physical health, cognitive-emotional well-being, and social engagement or support [4, 5]. There is an extensive range of HRQL instruments with demonstrated validity and reliability, and that serve as prognostic indicators for clinical outcomes [6, 7]. A separate lineage of research quantifies HRQL using a standardized utility value for a given medical condition, which is adjusted according to a specified time period of living with that condition (e.g. quality adjusted life year) [8].

In the Montreal Accord to Accelerate and Harmonize Patient-Reported Outcomes, Mayo et al. [2] noted two important issues concerning HRQL. First, it is common to find that multiple definitions of this construct are used in clinical settings. Second, it is challenging to specify what measures matter most to patients. This paper will revisit both issues. We will begin by mapping recent developments in HRQL assessment. We will then examine a representative sample of psychometric measures to identify core theoretical/philosophical themes that are embedded in these models of an HRQL state. These themes will be contrasted with patient reports where HRQL is described as an appraisal of a dynamic, adaptive process. We will suggest that it is advisable to extend the established definition of HRQL to accommodate insights from patient reports. HRQL will be defined as *an appraisal that evolves over time as it reflects and informs the self-regulatory process of adapting to dynamic changes in our health status.* The final section will revisit aspirational themes of HRQL processes that are evident in patient reports and this will be presented using a meta-philosophical approach that draws on the idea of philosophy as a way of living, as developed by Hadot [9–12] and others [13, 14]. We will argue that the Stoic modification of the ancient eudaimonic conception of well-being, presently recognized in HRQL literature, can make an important contribution to HRQL models. This paper will conclude by introducing an HRQL research agenda that is complementary to established HRQL assessments, and which focuses on self-reported, goal-directed processes that are initiated or maintained to promote individual HRQL and well-being. This agenda, we suggest, represents a novel, theoretically informed approach to HRQL assessment.

## HRQL in clinical settings and the encounter with construct pluralism

HRQL is an umbrella term [15] that assimilates multiple life domains that span bio-psycho-social dimensions as outlined by Engel [16]. A closer look at the above-noted HRQL definition [2] clarifies that it is a conceptual framework that can accommodate diverse operational definitions. Karimi and Brazier [17] trace the introduction of the term (HRQL) to the 1980’s where Kaplan and Bush spoke about it as a comprehensive and standardized measure of health status [18]. Interestingly, Kaplan recently published a retrospective appraisal where he commented that HRQL had become characterized by a diverse range of measures, and that many of these were not interchangeable due to theoretical and methodological divisions [19]. This comment echoed previous reviews where the goal of achieving consensus in HRQL theory and practice was seen as a primary challenge [20]. Alternatively, other reviewers have proposed whether it is feasible or preferable to strive for consensus with a unifying definition of health, where HRQL could be operationally defined by, or restricted to, a fixed set of common features [21].

### Opportunities for a common ground in HRQL theory

There is an historical initiative to establish a common theoretically informed methodology for HRQL assessments. The Wilson and Cleary taxonomy [22] was introduced in 1995 and it continues to play a constructive role in consolidating HRQL research, as evidenced by its influence on the Montreal Accord [2]. This approach has guided the development of a standardized template to define HRQL within bio-psycho-social domains of health [16]. In their systematic review of HRQL, Ojelabi et al. [23] report that the Wilson and Cleary taxonomy [22] is the most commonly cited HRQL framework in health research. Moreover, they also report moderate empirical support for its validity, with its core components accounting for up to 72% of the variance in patient-reported outcomes. This framework continues to evolve with adaptations such as the 2005 model by Ferrans et al. [24], that expanded the hypothesized life domains that account for HRQL. A recent systematic review of this version of the taxonomy reported that among 31 studies (pooled *n* = 62,281), 19 of 20 hypothesized associations between life domains and HRQL were supported [25]. Interestingly, this evidence was obtained from studies where a clinically applied theory or framework for health behavior or HRQL was referenced in only 16.5% of papers, while only 6% applied a theory to account for the constructs used in assessing HRQL. This suggests that it will be challenging to use findings from this research to systematically evaluate and improve upon current models of HRQL. A more recent adaptation of the Wilson and Cleary taxonomy has prepared this model for the digital transformation of health practice and research. Mayo et al. [2] modified it to incorporate patient reported indices of symptoms, function, and well-being, as well as objective physiologic indices (e.g. blood pressure, heart rate) that are now routinely obtained from digital/tele-monitoring.

A key challenge for building consensus in HRQL research is that new models are developed in a research environment where theories of health or HRQL are discussed in less than 17% of studies [20, 26, 27]. With the absence of papers that explicitly present or cite a theoretical framework for HRQL, there appears to be growing confusion about the construct that is being measured, as the same psychometric instruments are introduced in different articles as an index of quality of life, health status, or HRQL [17]. This begs the question of how theoretical innovations can be best introduced. To that end we will begin with a schematic summary of core theoretical/philosophical themes that are embedded in psychometric assessments and patient reports of HRQL.

## How do HRQL assessments compare with reports of persons with lived experience?

### Core themes in standardized HRQL assessment

Kelkar et al. [28] provided a “state of the art” systematic review of patient-reported outcomes for chronic heart failure (CHF). Table 1 provides a schematic summary of these HRQL assessments. The sampled questionnaires included patient-reported ratings of the degree to which CHF affected a spectrum of domains for living well: i.e. physical activities, activities of daily living, social activities, perceived impairment in emotional well-being, life satisfaction, psychological health, and symptoms of disease severity. From a philosophical perspective, this profile can be summarized as an index of how an individual’s HRQL appraisal is shaped by at least three foundational themes:

- *hedonic well-being*—i.e. a state marked by the absence of symptoms of physical discomfort or emotional distress, and/or elevated positive feelings and experiences; as sampled by self-ratings of shortness of breath, fatigue, worry, or depression [29, 30];
- *eudaimonic well-being*—i.e. the perception of flourishing in personal growth and happiness; as sampled by self-ratings for happiness or satisfaction with life [31, 32];
- *desire-satisfaction*—i.e. a state where desire is fulfilled by attaining a wide spectrum of objects or experiences to which we are attracted; as sampled by being able to engage in recreational activities, or in intimate/sexual relations [33, 34].

Inspection of the right column of Table 1 is a reminder that “…in today’s medical sciences the stand-in for well-being is *health-related quality of life*…” (Alexandrova, p .168 [35]). Indeed, the philosophical themes in Table 1 are similar to those evident in the World Health Organization definition of mental health. Here it is a state of well-being where an individual can cope with normal life stress (hedonic theme), work productively and fruitfully and make a contribution to their community (eudaimonic theme), and realize their abilities (desire-satisfaction theme) [36]. Additional philosophical themes might provide a more granular analysis of HRQL questionnaire items, but arguably the themes in Table 1 are satisfactory for a schematic summary. It is feasible to conceive of higher order interactions between the three philosophical themes for almost all of the questionnaire items: e.g. a positive rating for sleep could be associated with the fullfillment of a personally salient desire, while reflecting positive mood and affect, and a sense of accomplishment that contributes to personal flourishing.

**Table 1:**
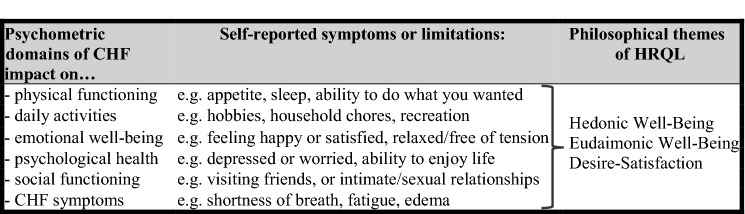
Schematic summary of HRQL questionnaire content adapted from the systematic review of Kelkar et al. [28]

### What persons with lived experience talk about when they talk about HRQL

Patient reports of HRQL highlight at least three core features that are distinct from operational definitions in psychometric assessments. First, these reports touch on multiple dimensions of life, each of which is meaningful insofar as it reflects an aspiration for living well (Table 2). These aspirations have been defined as life goals that are organized around intrinsic personal priorities that include (i) sustaining personal growth, (ii) participating in life events that are perceived as being meaningful, (iii) sharing time with a loved one, family, or friends, (iv) fulfilling social roles and responsibilities in which one is emotionally invested, or (iv) maintaining physical health and emotional well-being (cf. internal vs. external aspirations in self-determination theory [37–39]). Individuals diagnosed with CHF describe the impact of their medical condition as a profoundly undesirable life change [40, 41], that evokes a quest for existential support [42, 43], due to uncertainty about the future [40], loss of person [44, 45], social isolation [41, 45], vulnerability to acute distress or resignation [46, 47], variability in the will to live [46, 47], and the perception that life is precarious and death inescapable [47]. The scope of personal loss that is captured in these expressions highlights the difficulty of initiating an additional response noted in Table 2—i.e. *acceptance* of the existential reality of living with a chronic progressive medical condition [40, 41]. In short, impaired physical function or reduced social activity may correspond to an HRQL questionnaire item where it is categorized as a functional or social limitation, respectively. But from a patient-centered perspective, as illustrated in Table 2, its meaning as a marker of HRQL is derived from its impact on the ability to pursue aspirational life goals. The issue here is that the HRQL construct that is measured by questionnaires can differ qualitatively from the inferred meaning expressed in patient self-reports, despite similar (surface) item content.

**Table 2:** Health-related quality of life themes based on comments about lived experience with chronic heart failure (CHF) reported in qualitative research

Health-related quality of life themes and issues	Sample comments by persons with lived experience of chronic heart failure
Undesirable life changes: ability to pursue (individual or social) self-actualizing activities	• “Before HF I was dynamic, I used to run, to cook, but all of a sudden I had to stop” [40] • “…we don’t do activities together, pretty much across the board. …I think that’s probably been the hardest…appearing normal and knowing that I can’t do what everybody else can do, or what I should be able to do if I was a healthy version of me.” [41]
Quest for existential support: evidenced by understanding and acceptance of self, or connection to greater purpose/reality	• “[Talking with a palliative care nurse] is significantly meaningful, both in regards to medicine and that stuff but also in how one understands…how should I put it…the whole thing.”[42] • “…any time that you go for a procedure or you have a serious illness or something like that you’d want the chaplain to come by and have a prayer with you that would help” [43]
Uncertainty about the future: existential dread about the finiteness of life	• ‘Who knows how many days I have to live? …You live with this big question mark.’ [40] • “I always have something that reminds me that I am not well and makes me aware that anything can happen to me at any time...” [47]
Loss of person: capacity to pursue a self-determined life that is valued as an expression of self	• “All the things I used to like doing, I’m no(t) able to do anymore.” [44] • “I want a life. I haven't got one, I haven't got one at all, so what can you do about that?” [45]
Social isolation: loss of ability to connect with significant others	• “…there will be times, for example, (my family) won’t invite me to an event or something like that, thinking it’s too late, …[I] would be too tired…. Because they are super sensitive, sometimes they forget that sometimes we just want to be normal….” [41] • “I don’t meet people. The only people I see now is my carers. Otherwise I don’t see a soul. I don’t see anybody…..Oh very very lonely. Very very lonely”. [45]
Vulnerability to acute distress or resignation:	• “…it’s there all the time – fear – em – ‘Am I going to die under the next one?’ I hope I get over this fear part – that’s the worst part” [46] • “…seeing myself in this situation and thinking that I can do almost nothing by myself makes me feel very overwhelmed” [47]
Variability in the will to live	• “I feel that my usefulness on earth is finished now. I’m neither use nor ornament now really” [45] • “…living with CHF is horrible because all day long, I have the feeling of drowning, and that is very hard to bear…I would not mind leaving, right? Although I have never said it, sometimes I have thought about (gets emotional) even taking several boxes of medicines or throwing myself over a bridge…” [47]
Perception that present life (and what is fundamentally valued in it) is precarious, and death inescapable	• “…I will not live for many more years. Therefore, I have no economic or material ambitions because of what I have left of my life…. …the only thing I want is to be able to live a few more years to be able to enjoy my family for a longer time (gets emotional). That is my only desire…" [47] • “You have to put the soul to rest because you don’t know when death arrives, if today or tomorrow.” [40]
Acceptance: Affirmation of life as being meaningful and valued by fulfilling social roles or appreciating daily events	• “…I have a family, a daughter and a nephew, so I have to go on for them. Now I’m living the illness with patience and with the will to go on, and to go on with courage and strength” [40] • “…within seconds you can be on your way in an ambulance to the hospital. So you certainly appreciate the little things.” [41]

Second, patient-reported HRQL is characterized by *dynamic* change in health status. The word dynamic is italicized because the onset of acute CHF symptoms is often unanticipated, and this evokes elevated stress, anxiety, or depression [46, 48]. In the case of CHF, as in other chronic conditions (e.g. kidney disease [49] or cancer [50]), the sudden or gradual deterioration in health status arises from a complex network of bio-behavioral interactions where change cannot be readily attributed to a simple (linear) association with a causal factor. Moreover, efforts by health professionals to treat and support an individual with CHF are constrained by the unpredictable disease trajectory and poor discrimination of many prognostic models [51].

Third, HRQL implies a key element that was partially introduced in the previous paragraph: acceptance of uncertainty with each change in health status. Acceptance of these changes becomes more meaningful and challenging as the individual encounters a reality that is imposed by their progressive medical condition [40, 41]. CHF is characterized by a gradual or sudden deterioration in cardiac function, followed by a period of stability due to treatment. Next, a progressive downward trajectory in health status follows that is punctuated by episodes of acute symptoms or medical crises. These episodes are followed by temporary periods of stability that eventually become shorter and less stable as CHF progresses [52]. Surikova et al. [41] observed that patients with CHF viewed their condition as a journey marked by recurrent experiences of a *new normal* that was characterized by progressive limitations in their life as a result of changes in their health status: “…your life completely changes. And what is abnormal to other people is (now) normal to you” (p. 5). Ironically, acceptance of these limitations was reported to inspire a renewed appreciation for the “little things” in life (Table 2). This suggests one method through which self-reported HRQL may improve over time. This last observation is consistent with findings from a systematic review of coping behaviors in response to CHF. Active emotion-focused coping, which includes acceptance of the disease, use of humor, or engagement in spiritual practices was positively associated with HRQL outcomes [53].

Patient reports of HRQL reveal philosophical themes that are distinct from those embedded in conventional psychometric assessments. These reports describe HRQL as an adaptive process as opposed to a fixed state of well-being. In addition, the aspirations expressed by patients provide a clinically compelling reference that can support an initiative to extend our current definition of HRQL [2]. We suggest the following:

HRQL is an appraisal that evolves over time as it reflects and informs the self-regulatory process of adapting to dynamic changes in our health status.

We propose that this statement is complementary to Alexandrova’s definition of a mid-level theory [35]. To paraphrase, it refers to an appraisal of well-being or HRQL that is *relativized* as an expression of a given subject’s point of view about a particular person (self or other) within a specific social context. We suggest that the above process-based definition of HRQL refers to goal-directed activities that account for *how* HRQL appraisals become relativized by individuals, as they pursue a sense of “living well” within the parameters of their daily life. Further, we suggest that a process-based approach to HRQL is complementary to Mayo et al.’s definition [2]. But there remains a question of whether the philosophical theories that influence current definitions of HRQL, including hedonic, eudaimonic, or desire-satisfaction theories [31, 32, 34], are adequate to capture the dynamic nature of patient experience. In the space remaining we will discuss how we can address what’s missing within an organized theoretical framework.

### A meta-philosophical approach to HRQL

#### HRQL and philosophy as a* way of living*

The work of Pierre Hadot is distinct in uncovering the historical origins of a meta-philosophical movement where teachings and insights had a therapeutic function to promote philosophy as a *way of life* (PWL) [9]. Sharpe and Ure [14] recently detailed how this movement continues to speak to modern experience, as it stretches across major schools of philosophy from the Hellenistic period of Greece to the Imperial period of Rome, into philosophers of the renaissance (like Michel de Montaigne) and the modern period (like Friedrich Nietzsche). Donald Robertson [54, 55] has demonstrated how Stoic philosophy as a way of life informs the theory of well-being that is used as a therapeutic goal in cognitive behavioral therapy. In this paradigm, major schools of philosophy advocated the pursuit of a goal that represented the best form of life within an aspirational model of (what is now termed) psychological well-being and HRQL.

As noted above, psychometric measures of HRQL have been influenced primarily by a hedonic model of well-being (introduced by Aristippus, 435–356 BCE) and a eudaimonic model (introduced by Aristotle, 384–322 BCE). The desire-satisfaction model can also be traced to Aristotle’s theory of desire for the good [56]. But this raises the question of why we would limit our access to potential insights and methodologies for well-being and HRQL to those two philosophers? Recent research by Hadot on the idea of philosophy as a way of life has highlighted that in the ancient world, by far the two more influential and widespread philosophical conceptions of living well involved a revision of hedonism into the search for inner tranquility (*ataraxia*), in Epicurean philosophy, and a challenge to Aristotle’s conception of eudaimonism, in Stoicism, which positioned a good flow of life *(euroia biou)* as the goal [9–11]. Both of these theories place great emphasis on issues identified in the above-noted qualitative research findings which other models do not prioritize. Notably, they promoted self-examination and working through negative emotions in the face of adversity, and the need to come to peace with the realities of loss, pain, suffering, and mortality.

In what follows, we examine the credentials of the Stoic approach to well-being as a theoretical framework for a revised conception of HRQL as an adaptive process.

#### The Stoic approach to HRQL as *euroia biou* (good flow in life)

The Stoics promoted the “good life” with an account that rivaled the eudaimonistic ideas of Aristotle which have informed the contemporary concepts of flourishing and happiness [57]. The latter suggested that the principal component of a fulfilled life was living according to the virtues: strengths of character led by justice, courage, moderation, and wisdom. Nevertheless, he added that the potential to live in this manner was dependent upon “external goods” that were beyond an individual’s control, such as good birth, health, social connections, and physical attractiveness (*Nicomachean Ethics*, I, pp. 7–10) [58]. In contrast, the Stoics made the stronger claim that virtues are not only necessary, but sufficient for living a good and happy life [10, 13, 59]. This claim interconnects with a further distinguishing assertion that living well involves living in harmony with our human fellows and the larger cosmos, which was understood as an ordering principle, *Logos* [9, 10]. Zeno and subsequent Stoic philosophers referred to this experience as a “good flow of life”, *euroia biou* [59, 60].

Stoic virtue was developed as an embodied “wisdom” about how to achieve and sustain *euroia*, despite good or bad fortune. Its teaching served as a guide on how to choose what is naturally preferable, when we can, and to accept realities that are beyond our control without becoming avoidably distressed. It aimed to promote well-being using reasoning and reflection to control our impulses and to seek what is best, as in Aristotle’s ideal of eudaimonia. But in contrast to Aristotle, the cultivation of wisdom and virtue was viewed as possible for anyone, from rich to poor, young to old, as well as those facing physical illness, ill-fortune, or untimely death. In Stoicism, “external goods” were deemed “indifferent” (neither intrinsically good or bad) and therefore unnecessary for living well. This perspective democratized the goal of *euroia* as accessible to all, which is remarkably compatible with modern ideals [10, 61], including the self-reported aspirations of individuals coping with progressive decline in their health status [41, 53].

#### Congruence between stoic exercises and HRQL-associated processes

The ancient genre of Stoic consolations to the exiled and bereaved speaks directly to an effort to promote HRQL in a way that is distinct from eudaimonism, hedonism, or desire-satisfaction. The Stoic framework includes activities that were utilized to transform experiences of loss, grief, separation, and the reality of our mortality [14, 62], where the promotion of HRQL and well-being in response to these adversities was reconfigured as good flow in life (*euroia*).

Table 3 presents emergent themes for HRQL processes from Stoic exercises. These included *restoring agency* in the face of adversity. Stoics emphasized “the dichotomy of control”, which involves redirecting attention away from grief at the loss, or fear about the possible loss of what we cannot control, towards maximizing resources that are available for our response to these events. This strategy has been formulated within cognitive behavioral theory as cognitive-restructuring [54, 55]. It stresses that happiness resides in affirming how even in the midst of perilous events we can develop and express our personal dignity and agency [10].

**Table 3:** Self-guided exercises to promote well-being extracted from the Stoic and Epicurean schools of ancient philosophy, using the meta-philosophical paradigm of philosophy as a *way of life*

School	Prescribed exercises	Therapeutic goal	Health-related quality of life activity theme
Stoic, Epicurean	Meditation (memorisation and recollection of key principles)	Recollection & reorientation around fundamental values; reconnection with larger whole (contemplation)	Self-actualization, Connection with larger meaning
Stoic	Applying dichotomy of control	Reducing negative emotion, reconnecting with fundamental values and priorities	Regulating emotion Self-actualization
Stoic, Epicurean	Paying attention to thoughts, judgements	Promoting apatheia, ataraxia, absence of negative emotions	Regulating emotion Self-actualization
Stoic	Daily premeditation of adversity	Preparation for difficulties, promoting ataraxia/apatheia	Regulating emotion Self-related health care
Stoic, Epicurean	Meditation on mortality	Reconnecting with fundamental values and priorities, removing unnecessary fears	Self-actualization Connection with larger meaning
Stoic	Nightly examination of conscience	Reconnecting with fundamental values and priorities	Self-actualization Connection with larger meaning
Epicurean	Recollection of pleasures	Promoting stable pleasure, ataraxia	Regulating emotion, reducing distress
Stoic	Reframing experiences (view from above)	Reducing negative emotions, overcoming adversities, reconnecting with larger whole	Regulating emotion, reducing distress Self-actualisation Connection with larger meaning
Stoic	Training in endurance	Reducing negative emotions, preparing for adversities	Health-related self-care Physical vitality
Stoic	Moderating emotions	Reducing negative emotions, promoting more stable positive states (apatheia, ataraxia)	Regulating emotions, reducing distress
Stoic, Epicurean	Regular reading of inspirational texts	Reconnecting with fundamental values and larger whole	Education to promote well-being Self-actualisation
Stoic	Writing, journaling	Recollection & reorientation around fundamental values, reconnection with larger whole (contemplation)	Self-actualization Connection with larger meaning
Stoic	Fulfilling social duties	Reconnection with others, recollection of belonging in human community	Social affiliation Fulfilling social duties Connection to larger meaning
Stoic, Epicurean	Friendship, relationship with counselor/teacher	Cultivating stable pleasures, affirmation of belonging and worth, examination of self and decisions	Social affiliation

Adapted from Sharpe and Ure [14]

There is secondly an extensive Stoic literature on the theme of d*ecreasing emotional distress while regulating physical and affective well-being* by moderating or extirpating fear, anger, and distress. This practice followed the Stoic understanding of emotions, which were viewed as physical responses that are shaped by our beliefs. The implied logic is that emotional well-being and equanimity can be enhanced by addressing and transforming these beliefs to reflect a greater detachment from things one cannot control [63]. This exercise is similar to previously noted coping efforts of CHF patients, where acceptance of their “new normal” health status was associated with an appreciation of the “little things” in life, that may be more controllable [41].

A related third practice was to promote study and reflection aimed at decreasing emotional distress associated with bereavement, while increasing an existential awareness and acceptance of our common mortality [62]. This consolation literature reflected an aspiration of *living in the present moment*, where the future, and as such our mortality, was to be accepted as beyond our control and as part of the greater natural “Whole”, to savor the time that is available each day, and maximize levels of attention on present experiences (cf. Marcus Aurelius’ *Meditations)* [9, 12].

Fourth, *fostering social affiliation and fulfilling social roles* was a distinguishing feature of Stoic teaching, which is far more robust than in an Epicurean model. For the Stoics, we are social and rational animals. Justice is a virtue since others are fundamentally like us in their capacity to reason and to strive for virtue. All human sociability, they argue, begins in family life, in children’s dependency on care providers, and the natural affection of parents and adults for the young [64]. This bespeaks a natural obligation to take care of others and to demonstrate patience and restraint in social interactions. For the Stoics, actions undertaken for the good of others, including future generations, are not acts of self-abnegation, so much as acts of real kinship. This exercise is partially consistent with current evaluations of social functioning and support that contribute to psychometric measures of HRQL. However, the notion of enhancing HRQL by fulfilling social roles or responsibilities is not commonly addressed.

Finally, Stoic meditations on our mortality, our rational nature, and our role in society emphasized each individual’s *connection with a greater purpose*. This was expressed in teachings and meditations of how we participate in an organizing principle of the cosmos (*Logos*) [9, 12]. The goal of *euroia biou* was to live in harmony with this larger order, which includes birth and death, and sickness and health, as equally natural processes of life. This exercise is consistent with active emotion-focused coping that has been shown to improve HRQL [53].

#### An HRQL research agenda that is theoretically informed by PWL themes

This paper presents a novel initiative for an HRQL research agenda that can only be introduced here. We advocate the development of patient-reported assessments to identify goal-directed activities (processes) that are explicitly initiated or maintained to promote HRQL and well-being. PWL research has identified several goal-directed activity themes that need to be empirically evaluated for their association with self-reported HRQL. It follows that an objective that is complementary to current HRQL research is to establish whether self-reported goal-directed activities can be summarized by a set of prototypical categories, which in turn could be evaluated for their ability to mediate therapeutic changes in HRQL. To this point, a proof of concept study by Syed et al. reported that self-reported goal-directed activities among patients with CHF predicted 12-month ratings of HRQL [65].

## Conclusion

This paper began by examining how HRQL assessment fits within the HRQL research environment. We then compared core philosophical themes that emerge from a representative sample of HRQL questionnaires, and we juxtaposed these themes with patient-reported insights from qualitative research. This comparison highlighted a discordance between standardized, state-based definitions of HRQL from questionnaires, and aspirational features from patient reports that suggested a process-based model of HRQL. In our final section we noted that the concepts of hedonic and eudaimonic well-being, and desire-satisfaction have had an imposing influence on HRQL assessment. We then used a meta-philosophical approach (PWL) to introduce a novel theoretically organized framework that could address aspirational themes of HRQL. This included introducing components of Stoic philosophy that reflected a process-based approach to HRQL, as exemplified by the concept of *euroia biou (good flow of life)*. We noted that Stoic themes of well-being are associated with adaptive processes or activities that included efforts to (i) connect to a greater purpose, or (ii) accept adverse life events, while (iii) embracing opportunities to reaffirm the meaning and value of one’s life. A novel process-based HRQL research agenda was introduced with attention to the potential value of assessing self-reported, goal-directed activities that are initiated or maintained to promote individual HRQL. We suggest that the process-oriented approach to HRQL assessment outlined here has the potential to make a meaningful addition to current patient-reported outcome research.

## Data Availability

This theoretical review paper does not include novel data that could be shared, therefore a data availability statement is not applicable.
